# An updated genetic marker for detection of Lake Sinai Virus and metagenetic applications

**DOI:** 10.7717/peerj.9424

**Published:** 2020-07-17

**Authors:** Deborah D. Iwanowicz, Judy Y. Wu-Smart, Tugce Olgun, Autumn H. Smart, Clint R.V. Otto, Dawn Lopez, Jay D. Evans, Robert Cornman

**Affiliations:** 1 Leetown Science Center, U.S. Geological Survey, Kearneysville, WV, United States of America; 2 Entomology, University of Nebraska—Lincoln, Lincoln, NE, United States of America; 3 Northern Prairie Wildlife Research Center, U.S. Geological Survey, Jamestown, ND, United States of America; 4 Beltsville Agricultural Research Center, U.S. Department of Agriculture, Agricultural Research Service, Beltsville, MD, United States of America; 5 Fort Collins Science Center, United States Geological Survey, Fort Collins, CO, United States of America

**Keywords:** Lake Sinai Virus, *Apis mellifera*, *Halictus ligatus*, Pollinator, Metagenetics

## Abstract

**Background:**

Lake Sinai Viruses (LSV) are common RNA viruses of honey bees (*Apis mellifera*) that frequently reach high abundance but are not linked to overt disease. LSVs are genetically heterogeneous and collectively widespread, but despite frequent detection in surveys, the ecological and geographic factors structuring their distribution in *A. mellifera* are not understood. Even less is known about their distribution in other species. Better understanding of LSV prevalence and ecology have been hampered by high sequence diversity within the LSV clade.

**Methods:**

Here we report a new polymerase chain reaction (PCR) assay that is compatible with currently known lineages with minimal primer degeneracy, producing an expected 365 bp amplicon suitable for end-point PCR and metagenetic sequencing. Using the Illumina MiSeq platform, we performed pilot metagenetic assessments of three sample sets, each representing a distinct variable that might structure LSV diversity (geography, tissue, and species).

**Results:**

The first sample set in our pilot assessment compared cDNA pools from managed *A. mellifera* hives in California (*n* = 8) and Maryland (*n* = 6) that had previously been evaluated for LSV2, confirming that the primers co-amplify divergent lineages in real-world samples. The second sample set included cDNA pools derived from different tissues (thorax vs. abdomen, *n* = 24 paired samples), collected from managed *A. mellifera* hives in North Dakota. End-point detection of LSV frequently differed between the two tissue types; LSV metagenetic composition was similar in one pair of sequenced samples but divergent in a second pair. Overall, LSV1 and intermediate lineages were common in these samples whereas variants clustering with LSV2 were rare. The third sample set included cDNA from individual pollinator specimens collected from diverse landscapes in the vicinity of Lincoln, Nebraska. We detected LSV in the bee *Halictus ligatus* (four of 63 specimens tested, 6.3%) at a similar rate as *A. mellifera* (nine of 115 specimens, 7.8%), but only one *H. ligatus* sequencing library yielded sufficient data for compositional analysis. Sequenced samples often contained multiple divergent LSV lineages, including individual specimens. While these studies were exploratory rather than statistically powerful tests of hypotheses, they illustrate the utility of high-throughput sequencing for understanding LSV transmission within and among species.

## Introduction

Lake Sinai Viruses (LSV) are single-strand RNA viruses first detected in metagenomic investigations of honey bees (*Apis mellifera*) (Runckel et al., 2011; Cornman et al., 2012). They produce no described disease syndromes in honey bees despite occasionally high prevalence and abundance in managed colonies (Runckel et al., 2011; Cavigli et al., 2016; Traynor et al., 2016; Glenny et al., 2017). Nonetheless, high rates of LSV infection are likely detrimental to honey bee colonies and could modulate the dynamics of co-occurring pathogens (Moreno-García et al., 2014; Traynor et al., 2016; Glenny et al., 2017; Schwarz, Huang & Evans, 2015). Suggestive correlations with colony health have been reported in some studies (Cornman et al., 2012; Daughenbaugh et al., 2015) but not all (e.g., Glenny et al., 2017). Whether LSVs have a significant impact on other insect populations also remains unknown.

The lack of characteristic disease symptoms has led to a dependence on molecular diagnostics for LSV surveillance. These surveys have suggested a cosmopolitan distribution of LSV without obvious geographic structuring of variants at continental scales (Bigot et al., 2017). LSV has been detected in several other hymenopteran species (Ravoet et al., 2014; Parmentier et al., 2016; Dolezal et al., 2016; Bigot et al., 2017) as well as the honey bee pathogen *Varroa destructor* (Acaridae) (Daughenbaugh et al., 2015), although to date replication intermediates have been demonstrated only in *A. mellifera* (Daughenbaugh et al., 2015). LSV abundance has been found unrelated to (Glenny et al., 2017) or negatively related to (Traynor et al., 2016) *V. destructor* infestation levels, indicating that the latter is not likely an important vector of LSV. LSV does not show codon co-evolution with honey bees (Cornman, 2019), as do some other RNA viruses (Chantawannakul & Cutler, 2008), and the extent of their host range remains unclear.

Molecular surveys of LSV prevalence have been complicated by the high diversity of LSV haplotypes. For example, protein divergence between the first LSV1 and LSV2 accessions reported by Runckel et al. (2011) is 20% at the polymerase “open reading frame (ORF)”. Subsequent work has confirmed that these two variants represent distinct and cosmopolitan phylogenetic clusters, but diverse sister lineages also exist and may have been under-represented by PCR-based surveys (Cornman, 2019). Indeed, single colonies and even individual bees can harbor multiple divergent lineages (Ravoet et al., 2015). Primers used for genetic detections have to date not shown sensitivity to the full range of known variants, and typically target a specific phylogenetic cluster to the exclusion of others (e.g., Runckel et al., 2011; Ravoet et al., 2014; Cavigli et al., 2016; Daughenbaugh et al., 2015; Traynor et al., 2016; Dolezal et al., 2016).

Here we report modestly degenerate primers that amplify a 365 nt stretch of the viral polymerase ORF. The primers match all known variants of LSV reported in a recent survey of LSV variation (Cornman, 2019). We tested the primers on three sample sets: (1) honey bees pooled at the apiary level from the west and east coasts of North America; (2) individual specimens collected while foraging on cultivated landscapes and road sides in the vicinity of Lincoln, Nebraska, including honey bees, native bees, and other pollinating insects; and (3) honey bees pooled at the colony level from North Dakota apiaries. The latter sample set was further divided into abdomen and thorax tissue pools to examine whether LSV variants were structured by tissue. Samples testing positive in these three sample sets were then assessed using metagenetic sequencing to evaluate the utility of the primers to partition complex viral mixtures.

## Materials & Methods

### Sampling

No new sampling occurred for this study. Sites used for sampling are listed in the cited previous work. These consisted of both our own institutional sites as well as private and public lands. Private property was accessed with the expressed verbal permission of landowners and beekeepers. The “U.S. Department of Agriculture (USDA)” sample set represents California (CA) and Maryland (MD) apiaries sampled in April 2018 following the methods of Traynor et al. (2016). CA colonies were situated in San Joaquin County, CA (36.6814, −120.5293) at time of sampling but had been relocated approximately one month prior from Washington state for commercial pollination. MD samples were collected from research colonies of the Bee Research Laboratory, US Department of Agriculture—Agricultural Research Service in Beltsville, MD (39.03265, −76.8762). Four CA samples and three MD samples testing positive for LSV2 with the approach of Traynor et al. (2016) (which used LSV2-specific primers designed by Runckel et al. (2011) and fluorescence-based detection of their amplification) and four CA and three MD samples testing negative by that protocol were re-evaluated with the new primers described herein. Each sample consisted of 50 bees collected from frames, from which RNA was extracted by bulk homogenization and cDNA generated by a combination of oligo(dT) and random hexamer priming. The “University of Nebraska - Lincoln (UNL)” sample set represents individual pollinators and other florally co-occurring insect specimens from Nebraska, collected as described in Karacoban (2018). Sites targeted distinct land-use types that support pollinating insects, including public gardens, agricultural areas, and road sides in the vicinity of Lincoln, Nebraska, with collections spanning the spring and summer of 2017 and 2018. Individual foraging honey bees, wild bees, wasps, beetles, ants and true bugs were netted and stored individually in vials at eight sites across an urban-rural land use gradient (bounding box [−96.8160, 40.7040, −95.9160, 41.2340]). The “U.S. Geological Survey (USGS)” sample set represents hives from multiple apiaries in the Prairie Potholes region of North Dakota (bounding box [−102.1948, 46.0885, −97.7289, 48.4146]), which have been described previously (Cornman et al., 2015). Twenty bees collected from frames were dissected to separate abdomen and thorax (wings and legs were removed). Abdomen and thorax tissues were homogenized separately so that each sampling event was represented by two tissue-specific cDNA preparations. For both the UNL and USGS sample sets, cDNA was prepared using the Applied Biosystems High Capacity cDNA Reverse Transcription Kit (Applied Biosystems) following manufacturer instructions.

### Assay amplification conditions

The LSV assay primers are described in the Results. Amplifications were initially performed in 25 µL volumes on a BioRad thermocycler using 0.15 µM of each primer, 1 µL of the initial amplification product, and Promega GoTaq Green Master Mix following manufacturer recommendations. The thermocycling program included an initial disassociation step of 98 °C for 3 min, followed by 36 cycles of 45 s at 98 °C, 30 s at 60 °C, and 1.5 min at 72 °C. Products were subjected to a final extension at 72 °C for 5 min and then held until collection at 12 °C. Amplification products were visualized by electrophoresis of 5 µL of reaction product and a 100 bp ladder through a 1.2% agarose gel (LabForce Agarose LE, Thomas Scientific) stained with GelRed (Thomas Scientific) at 100 V for 45 min. PCR products were cleaned with the Qiagen Qiaquick Purification kit and quantified using the Qubit dsDNA HS Assay Kit (Life Technologies). Samples were diluted in Nuclease Free Water (Qiagen) to a final concentration of 5 ng/µL.

A second round of amplifications was performed for the USDA sample set with the new LSV primers using the quantitative reverse transcription PCR (RT-qPCR) methodology of Traynor et al. (2016). The annealing temperature was adjusted to 60 °C and template levels were scored as present if fluorescence levels exceed the critical threshold (Ct) prior to 40 cycles of amplification. This allowed a direct comparison of amplicon detection by agarose gel and by fluorescence imaging software. Amplicons from the latter reactions were purified and Sanger-sequenced to confirm their identity, but as they were performed after the metagenomic sequencing run they were not included in the metagenetic analysis.

### Illumina amplicon sequencing

For samples classified as positive by visualization in agarose, 1 µL of product from positive reactions was used to generate amplicon sequencing libraries as directed by Illumina’s 16S Metagenomic Sequencing Library Preparation Protocol (Illumina). The LSV primers were modified with the overhangs specified in the protocol and dual Nextera XT indexes (Illumina Inc.) were incorporated following manufacturer’s instructions. Libraries were quantified with the Qubit dsDNA HS Assay Kit (ThermoFisher Scientific). DNA size spectra of completed libraries were in the range of 446–501 bp, as determined with the Agilent DNA 100 Kit on an Agilent 2100 Bioanalyzer (the expected library size is 492 bp after all Illumina adapters for dual-index sequencing are appended). Indexed libraries were diluted to 4 nM using 10 mM Tris pH 8.5 and pooled. A final 6 pM preparation was created with a 15% PhiX control spike and run on a MiSeq Reagent Kit v3 (600 cycles). A single sample (UNL.VT.250) was processed in triplicate to assess technical variation, encompassing all library preparation and sequencing steps from the initial PCR but not the prior steps of RNA extraction and cDNA preparation.

### Clustering and quantification of variants

Read pairs were trimmed of adapter sequences and low-quality bases (Phred score < 10) with BBduk (Bushnell, 2020), after which reads less than 250 nt in length were discarded. Intact read pairs were then merged with BBduk using the “loose” parameter setting. The primer sequence was not removed at this stage to facilitate clustering and alignment into operational taxonomic units (OTUs). Merged reads were clustered by sample using the “cluster_size” option of vsearch (Rognes et al., 2016) at 98% identity (as defined by option “1” of that program). Singleton clusters and cluster representatives with ambiguity characters were discarded. Sample-level clusters were then pooled and re-clustered using the same parameters to produce an initial set of 3,109 OTUs. No nucleotide error-based denoising was performed at this stage, because OTUs were later denoised and dereplicated based on protein translation as described below. Protein-level denoising removes OTU candidates of questionable biological relevance that might be retained by nucleotide denoising (i.e., OTUs differing only by common synonymous substitutions or that contain frameshift or nonsense mutations). On the other hand, protein-level denoising eliminates synonymous nucleotide variation that could be functionally significant for other reasons, such as via RNA folding or RNA-protein interactions, or which could otherwise be useful for studying viral history or demography. Thus, the denoising method should be considered in light of study objectives.

OTU representatives were codon aligned with MAFFT (Katoh et al., 2002) using default settings and the alignment manually edited in BioEdit (Hall, 1999). Sequence 5′ or 3′ of the primer positions was trimmed from the alignment and OTUs with gaps at either terminus, or with interior gaps exceeding 6 nt in length, were removed. Sequences were then translated to identify OTUs with stop codons or frameshifts, which were also discarded. OTUs were then dereplicated at the protein level using cd-hit (Fu et al., 2012), resulting in 562 final OTUs. These OTUs were examined for chimeras using the “uchime_ref” command in vsearch with the sequences shown in File S1 as the reference database, but no chimeras were detected with default settings.

To generate sample-level counts, merged reads were mapped to the translated final OTUs using BLASTX with default settings, retaining only the top match. Prior to mapping, the primer regions were removed from the OTUs, excepting the invariant first position of the reverse primer to preserve the reading frame. Alignments were considered invalid if they were not 104-105 codons in length or had more than three mismatch or gap positions combined. OTUs with less than 10 total counts were not included in the compositional analysis. OTUs that were counted only once in a sample were considered potential sample crosstalk and converted to zero for that sample. Samples with fewer than 1,000 total counts were excluded from compositional analyses.

The base-level sequencing error rate was estimated independently by mapping merged reads (minimum length of 363 nt) to the primer-trimmed positive control sequence, using the “fast” and “local” settings in bowtie2 (Langmead & Salzberg, 2012). Mismatch frequency was calculated from the resulting alignment with the Alfred package (Rausch et al., 2018).

### Compositional analysis

A neighbor-joining dendrogram of high-frequency OTUs was created in MegaX (Kumar et al., 2018) using the JTT protein-distance matrix and assuming uniform rates. High-frequency variants were defined as OTUs with a compositional abundance greater than 10% in at least one sample. The dendrogram was used to identify OTUs that clustered closely with LSV1 or LSV2, or were divergent in character, not to test a specific phylogenetic hypothesis. Pairwise protein differences among OTUs were calculated with MegaX and then parsed with perl, from which we calculated a weighted distribution of amino-acid changes relative to the most common OTU. The code for this calculation is included in File S2. UniFrac distances (Lozupone & Knight, 2005) between sample pairs were calculated with phyloseq (McMurdie & Holmes, 2013), for which the underlying OTU distances were derived using neighbor joining and the JTT distance matrix in MegaX. An unrooted, sample-level neighbor-joining tree was estimated from the UniFrac distances using ape (Paradis, Claude & Strimmer, 2004). In accordance with U.S. Geological Survey policy, the data and metadata associated with this study have also been made available in an approved repository (Cornman, Iwanowicz & Otto, 2020).

**Table 1:** Characteristics of the Lake Sinai Virus (LSV) primer assay used in this study. Adapter sequences for Illumina MiSeq are italicized. Degenerate sites represented by IUPAC ambiguity codes are underlined.

Primer name	Sequence	Predicted Tm (°C)
LSV-for	CKTGCGGNCCTCATTTCTTCATGTC	58.1–62.2
LSV-rev	CATGAATCCAAKGTCAAAGGTRTCGT	55.5–59.2
Miseq-LSV-for	*TCGTCGGCAGCGTCAGATGTGTATAAGAGACAG*CK TGCGGNCCTCATTTCTTCATGTC	70.2–71.5
Miseq-LSV-rev	*GTCTCGTGGGCTCGGAGATGTGTATAAGAGACAG* CATGAATCCAA KGTCAAAGGTRTCGT	69–70.2

## Results

### Primer development and application to surveys

The aligned RNA-dependent RNA polymerase sequences used to identify primer candidates were obtained from Cornman (2019) and trimmed as shown in File S1, excluding the hypervariable region identified in that study. We manually selected a single primer set that required two degenerate sites in each primer to match all variants in the alignment (Table 1). Degenerate sites do not occur within four positions of the 3′ terminus of either primer. The maximum difference in predicted Tm among degenerate variants was 4.1 °C. The forward primer (LSV-for) overlaps substantially with that used by Dolezal et al. (2016) but the reverse primer (LSV-rev) site begins 155 nt downstream of the reverse primer used by those authors. Note that in the present work we have eschewed LSV identifiers other than LSV1 and LSV2, because existing nomenclature does not encompass the full range of LSV variants (see Cornman, 2019) and definitive classification methods are lacking. We continue to use LSV1 and LSV2 as these labels were established simultaneously in the literature by their original discoverers (Runckel et al., 2011), are consistently identified in phylogenetic analysis as homogeneous clusters well diverged from each other, are in wide usage and recognized as distinct taxa in NCBI databases, and the presence of one or both is nearly ubiquitous in surveys. This does not imply that LSVs other than LSV1 and LSV2 are excluded from File S1 (they are not) and the selected primers do match other known LSV lineages.

The primers successfully amplified a control sequence identical to the LSV1 sequence of (Runckel et al., 2011) (positions 2155–2519 of reference sequence HQ871931.2), which was also used as a positive control library in the sequencing run, described below. In the USDA sample set, the four sample pools from CA that previously tested positive yielded the target amplicon. However, all MD samples were judged negative when visualized in agarose, including the three previously classified as LSV2 positive. These MD LSV2-positive samples were also judged negative in agarose when the amplification was repeated with the LSV2-specific primers of Runckel et al. (2011), suggesting that either these cDNAs had decayed in storage or the negative results were attributable to scoring method rather than primer pair. Re-analysis with primers LSV-for and LSV-rev (Table 1) but using the RT-qPCR approach of Traynor et al. (2016) confirmed amplification in the three MD LSV2-positives as well as in two of the seven LSV2-negative samples from CA and MD. Amplicons migrated in agarose at the expected size range and without smearing or extra bands (Fig. S1). A subsample of bands was purified and the target amplicon confirmed by Sanger sequencing (File S3).

In the UNL multispecies sample set, 18 bee species (Apiodea) were tested, among 25 insect species total, although for most species only one or a few specimens were available (Table 2). For *A. mellifera*, nine of 115 specimens yielded positives, or 7.8%. The native bee species *Halictus ligatus* yielded positives in four of 63 specimens, or 6.3%. No other positive samples were detected. It bears emphasizing that detection by this method does not demonstrate replication and that other RNA viruses classically associated with honey bee (e.g., Deformed wing virus and Sacbrood virus) were also detected in species other than honey bee, including *H. ligatus* (Karacoban, 2018). In the USGS sample set (20 individuals per pool), 13 of 24 colony-level samples were positive. For eight of the 13 positives, amplification was detected from only one of the paired tissues. There were slightly fewer detections with thorax tissue (eight of 24, 33%) than with abdomen tissue (10 of 24, 41.7%), consistent with the higher average LSV2 copy numbers estimated by Daughenbaugh et al. (2015) for the latter tissue.

**Table 2:** Lake Sinai Virus (LSV) detections rates by end-point PCR. Detection rates by sample group and species are presented as the ratio of samples testing positive to the total number of samples tested.

Sample set	Species	Order	Type	LSV detection rate (positive/total tested)
USDA	*Apis mellifera*	Hymenoptera	Pool of 50 workers, CA, LSV2 positive	4/4
USDA	*Apis mellifera*	Hymenoptera	Pool of 50 workers, CA, LSV2 negative	1/4
USDA	*Apis mellifera*	Hymenoptera	Pool of 50 workers, MD, LSV2 positive	3/3
USDA	*Apis mellifera*	Hymenoptera	Pool of 50 workers, MD, LSV2 negative	1/3
USGS	*Apis mellifera*	Hymenoptera	Pool of 20 workers, thorax tissue	8/24
USGS	*Apis mellifera*	Hymenoptera	Pool of 20 workers, abdomen tissue	10/24
UNL	*Agopostemon splendens*	Hymenoptera	Single whole specimen	0/4
UNL	*Apis mellifera*	Hymenoptera	Single whole specimen	9/115
UNL	*Bombus griseocollis*	Hymenoptera	Single whole specimen	0/22
UNL	*Bombus impatiens*	Hymenoptera	Single whole specimen	0/17
UNL	*Bombus pennsylvanicus*	Hymenoptera	Single whole specimen	0/3
UNL	*Cerotoma trifurcata*	Coleoptera	Single whole specimen	0/1
UNL	*Chauliognathus pennsylvanicus*	Coleoptera	Single whole specimen	0/4
UNL	*Cosmopepla lintneriana*	Hemiptera	Single whole specimen	0/1
UNL	*Formica pallidefulva*	Hymenoptera	Single whole specimen	0/3
UNL	*Halictus confusus*	Hymenoptera	Single whole specimen	0/1
UNL	*Halictus ligatus*	Hymenoptera	Single whole specimen	4/63
UNL	*Hylaeus annulatus*	Hymenoptera	Single whole specimen	0/1
UNL	*Hylaeus verticalis*	Hymenoptera	Single whole specimen	0/7
UNL	*Megachile brevis*	Hymenoptera	Single whole specimen	0/1
UNL	*Megachile mendica*	Hymenoptera	Single whole specimen	0/1
UNL	*Megachile pruina*	Hymenoptera	Single whole specimen	0/1
UNL	*Megachile rotundata*	Hymenoptera	Single whole specimen	0/1
UNL	*Melissodes bimaculatus*	Hymenoptera	Single whole specimen	0/2
UNL	*Melissodes communis*	Hymenoptera	Single whole specimen	0/2
UNL	*Melissodes comptoides*	Hymenoptera	Single whole specimen	0/1
UNL	*Melissodes desponsus*	Hymenoptera	Single whole specimen	0/3
UNL	*Popillia japonica*	Coleoptera	Single whole specimen	0/2
UNL	*Sphex jamaicensis*	Hymenoptera	Single whole specimen	0/1
UNL	*Sphex pensylvanicus*	Hymenoptera	Single whole specimen	0/3
UNL	*Xylocopa virginica*	Hymenoptera	Single whole specimen	0/1

### Metagenetic characterization of LSV diversity

All positive samples from the USGS and UNL sample sets (18 and 13, respectively) and all four CA LSV2-positive samples from the USDA sample set were included in the metagenetic sequencing steps, but with the expectation that libraries based on weak initial amplifications might fail. We did not attempt to re-amplify weak bands for sequencing out of concern that the resulting compositions would be less representative of the true composition. The additional positives of the USDA sample set identified by qRT-PCR were generated after the metagenetic sequencing run and thus no metagenetic data were generated for those samples. A complete replicate of one sample (UNL.VT.250) and a positive and negative control were also included. A second replicate of UNL.VT.250 was included on an otherwise unrelated sequencing chip to evaluate compositional variation across runs.

After amplification with the adapter-appended primers (Table 1) and Nextera indexed sequencing primers (see Methods), 10 of the 35 biological samples were judged (either by visualization in agarose or library quantification) inadequate to warrant loading on the sequencing chip. After sequencing, eight additional libraries with fewer counts than the negative control were considered failed libraries. Fourteen of the remaining 17 sample libraries and the positive control library exceeded the 1,000 read threshold we imposed for quantitative analysis (Table 3).

**Table 3:** Amplicon sequencing yield for samples positive for Lake Sinai Virus (LSV). The number of sequence reads mapping to an LSV operational taxonomic unit (OTU) for each sequenced sample. Samples with fewer than 1,000 total counts were excluded from compositional analysis of OTU abundance. Sample libraries producing fewer counts than the negative control were considered failed libraries and are not shown. The negative control sample was a water blank and the positive control sample was a known LSV1 sequence.

Sample ID	Counted LSV reads	Source	Species	Description	State	Collection date	Excluded from analysis?
USGS.CO.135ABD	1571	USGS	*Apis mellifera*	20 foragers, abdomen, single managed colony	ND	09/17	
USGS.CO.225ABD	11626	USGS	*Apis mellifera*	20 foragers, abdomen, single managed colony	ND	09/17	
USGS.CO.225THX	1864	USGS	*Apis mellifera*	20 foragers, thorax, single managed colony	ND	09/17	
USGS.CO.226ABD	24	USGS	*Apis mellifera*	20 foragers, abdomen, single managed colony	ND	09/17	Yes
USGS.CO.226THX	6428	USGS	*Apis mellifera*	20 foragers, thorax, single managed colony	ND	09/17	
USGS.CO.236ABD	55211	USGS	*Apis mellifera*	20 foragers, abdomen, single managed colony	ND	09/17	
USGS.CO.236THX	4515	USGS	*Apis mellifera*	20 foragers, thorax, single managed colony	ND	09/17	
UNL.H01.008	77	UNL	*Apis mellifera*	foraging individual, whole body, urban garden	NE	05/17	Yes
UNL.H01.009	74344	UNL	*Apis mellifera*	foraging individual, whole body, urban garden	NE	05/17	
UNL.H15.010	41797	UNL	*Apis mellifera*	foraging individual, whole body, urban garden	NE	07/17	
UNL.H23.001	22	UNL	*Apis mellifera*	foraging individual, whole body, urban garden	NE	05/17	Yes
UNL.LG.121	32956	UNL	*Halictus ligatus*	foraging individual, whole body, urban garden	NE	08/18	
UNL.VT.250	39605	UNL	*Apis mellifera*	foraging individual, whole body, urban garden	NE	08/18	
UNL.VT.250.replicate1	39349	UNL	*Apis mellifera*	foraging individual, whole body, urban garden	NE	08/18	
UNL.VT.250.replicate2	17144	UNL	*Apis mellifera*	foraging individual, whole body, urban garden	NE	08/18	
USDA.B5	67675	USDA	*Apis mellifera*	50 workers, abdomen, pooled across multicolony apiary	CA	04/18	
USDA.C11	73282	USDA	*Apis mellifera*	50 workers, abdomen, pooled across multicolony apiary	CA	04/18	
USDA.C2	36774	USDA	*Apis mellifera*	50 workers, abdomen, pooled across multicolony apiary	CA	04/18	
USDA.G9	45562	USDA	*Apis mellifera*	50 workers, abdomen, pooled across multicolony apiary	CA	04/18	
POSITIVE	52820	Positive control	NA	Sequence matches accession HQ871931.2	NA	NA	
NEGATIVE	11	Negative control	NA	Water added to reaction instead of DNA	NA	NA	Yes

**Notes.**

USGS U.S. Geological Survey, Northern Prairie Wildlife Research Center UNL University of Nebraska, Lincoln USDA U.S. Department of Agriculture, Agricultural Research Service, Bee Research Laboratory, Beltsville, MD NA not applicable

A total of 562 OTUs resulted from the clustering and filtering procedure, of which 518 had ten or more counts in the combined data set (OTU sequences are provided in File S4 and raw counts are included in File S5). The technical replicates of sample UNL.VT.250 in the same run had a Pearson correlation of 0.995, whereas the mean pairwise Pearson correlation declined slightly across runs (0.979, Fig. S2). This sample had the highest proportion of low-frequency OTUs of those analyzed. The base-level sequencing error rate was estimated to be 1.4% for the LSV positive control, whereas it was reported to be 3.6% by the MiSeq software based on the internal standard for the run.

**Figure 1:**
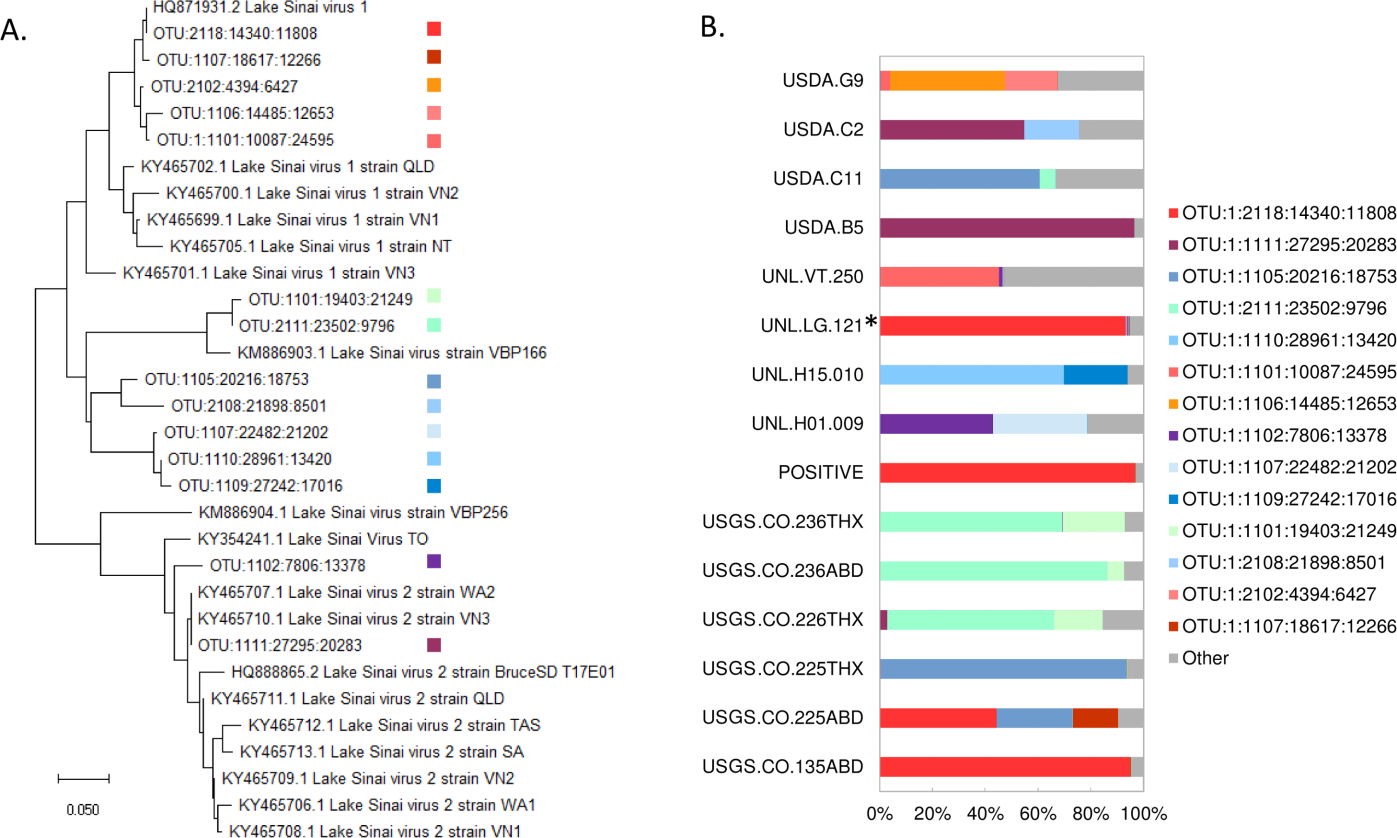
Phylogenetic relationships of high-frequency OTUs to public LSV accessions and their relative proportions in samples. (A) A neighbor-joining amino-acid phylogenetic tree depicting the diversity of high-frequency OTUs recovered by metagenomic sequencing. High-frequency OTUs were defined as comprising more than 10% of reads in at least one sample. The tree is based on a 105-residue alignment of the translated and primer-trimmed amplicon, using the JTT amino-acid distance matrix and ignoring site variation, and was created with MegaX (Kumar et al., 2018). The numerical code serves only as an OTU identifier. (B) The relative composition of each sample used in the quantitative analysis. High-frequency OTUs are color-coded as shown in panel A, in which phylogenetic clusters are designated by similar colors. Counts not assigned to a high-frequency allele are aggregated under the category “Other”. The asterisk-marked sample is *Halictus ligatus*. All other biological samples are *Apis mellifera* and the positive control sample is indicated.

**Figure 2:**
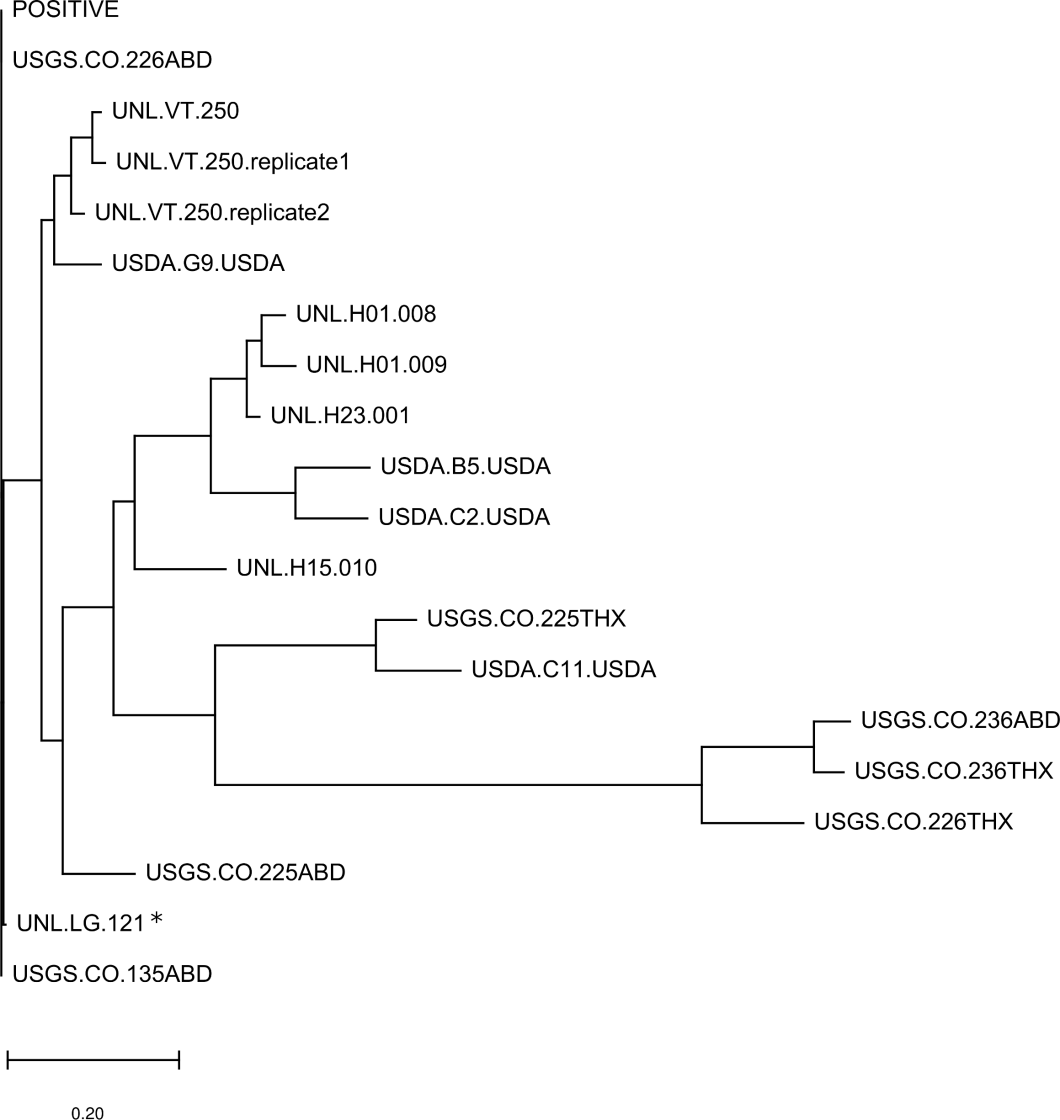
A sample-level dendrogram shows limited clustering by sample group. The dendrogram is based on the UniFrac distance measure and was constructed by neighbor joining. The phylogenetic component of UniFrac distance is based on protein distance for the 105-residue alignment of the primer-trimmed and translated LSV amplicon, as described in the methods. The asterisk-marked sample is *Halictus ligatus*. All other biological samples are *Apis mellifera* and the positive control sample is indicated.

Overall, fourteen high-frequency OTUs were identified, operationally defined as comprising more than 10% of the assigned reads in at least one sample. Half of these major OTUs clustered with LSV1 or LSV2 and half were intermediate lineages not well represented by existing accessions (Fig. 1A). Note the dendrogram is intended to represent the phylogenetic diversity of sequences recovered but should not be construed as strong evidence of the branching of LSV lineages (see Cornman, 2019 for a recent genomic analysis of LSV diversity). The relative abundance of major OTUs in each sample is illustrated in Fig. 1B. A sample-level dendrogram based on the UniFrac distance measure across all OTUs is shown in Fig. 2. We conclude from these results that LSV variation is not strongly structured by sample group and all groups harbor multiple LSV clades (Fig. 1A).

**Figure 3:**
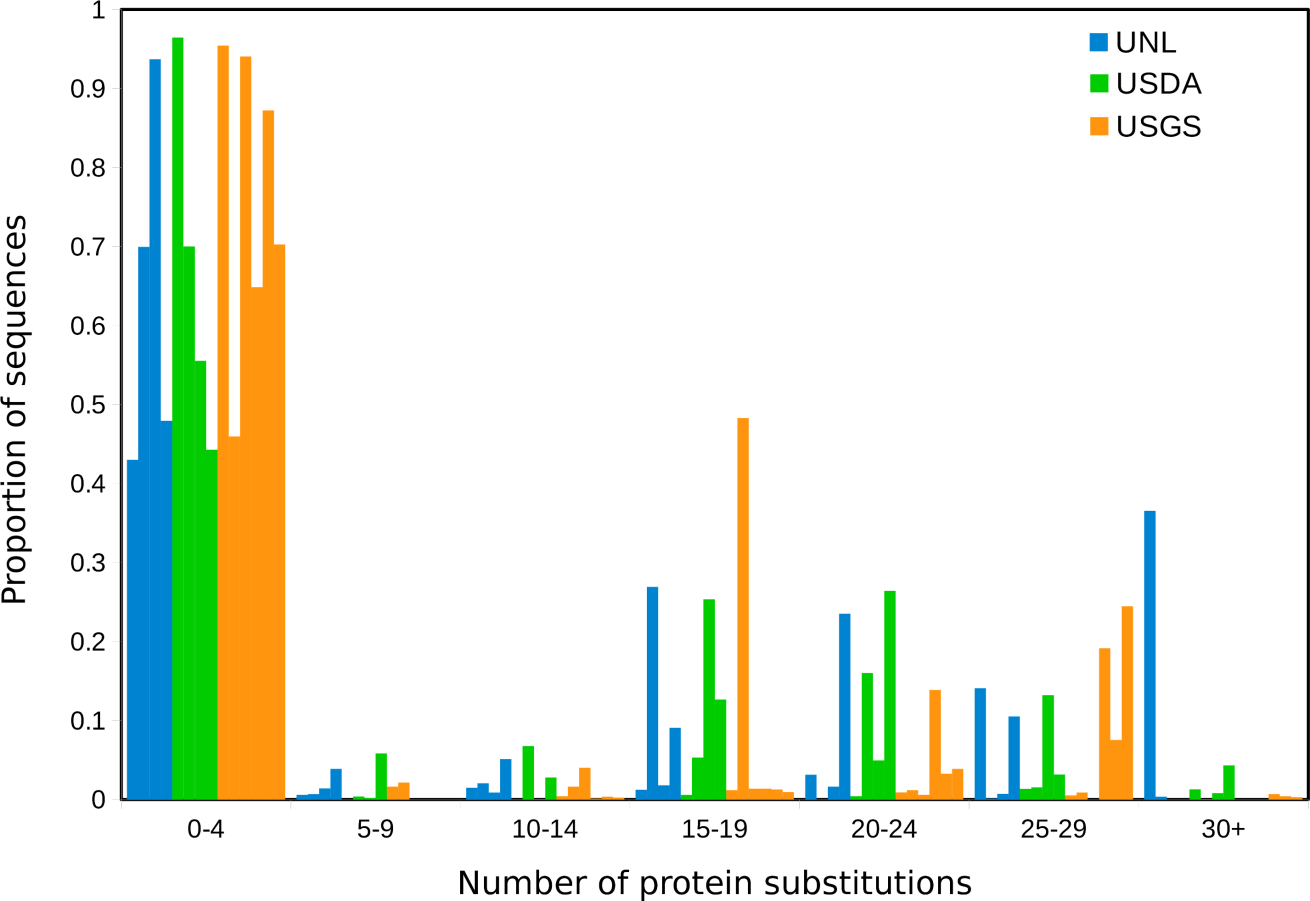
Co-occurring LSV sequences are diverse, even within individual specimens. The horizontal axis represents bins of protein distance (number of pairwise protein differences in the 105-residue alignments of primer-trimmed and translated amplicons) between the most-frequent OTU in each sample and all other OTUs of that sample. The vertical axis represents the proportion of sample reads in each bin. Samples are color-coded by sample set rather than individually, to highlight group-level distributions.

For each sample, we determined the number of protein differences between the most abundant OTU and all other OTUs, weighted by their relative abundance, and then binned these distances by increments of five substitutions (Fig. 3). Based on this calculation, LSV OTUs were often highly diverged from the dominant OTU of each sample, implying coinfection with diverse strains rather than mutation or sequencing noise. Indeed, individual specimens in the UNL sample group appeared to contain more divergent LSV haplotypes than did pools of 20 or 50 bees from single hives (the USGS and USDA sample groups, respectively). However, it is not possible to determine from these data whether this pattern differs from random assortment (i.e., as a function of the unknown frequency of LSV lineages in the environment).

## Discussion

Our first objective in this study was to increase the diversity of LSV sequences detectable by a single end-point PCR assay. Our results demonstrate that both the LSV1 and LSV2 phylogenetic clusters can be detected as well as diverse sister lineages. The primers have minimal degeneracy and no degenerate sites occur near the 3′ terminus of either primer, suggesting that amplification bias is unlikely to be large. However, we have not yet sequenced diverse mock communities to confirm this. While the amplicon is larger than that traditionally used in qPCR experiments, whether total LSV can be accurately quantified with these primers remains to be tested. Regardless, quantitative analysis of fluorescence curves (e.g., estimation of Ct values and amplicon melting temperature) was more sensitive in our hands for LSV detection than visualization in agarose. As two of the three sample sets analyzed were archived cDNA preparations, we did not use a strand-tagged reverse transcription PCR (RT-PCR) assay to confirm the negative-strand replication intermediate (see Daughenbaugh et al., 2015, for example).

The second objective of this study was to evaluate metagenetic sequencing of the primer-defined locus on a variety of sample types. We investigated three sample sets that represented spatial-, tissue-, and host-level variation. The number of strong positive samples available for metagenetic sequencing was modest but did not reveal strong geographic structure in aggregate (i.e., by UniFrac distance). However, high-frequency OTUs were predominantly LSV1 or phylogenetically intermediate lineages, with LSV2 less represented and rare in the USGS samples. In comparison, Cavigli et al. (2016) initially found LSV2 more abundant than LSV1 or other variants in managed honey bees in a single season of almond pollination in California. However, a detailed breakdown of viral abundance by colony (Glenny et al., 2017) showed both major LSV variants persisting at comparable levels in this almond-pollination setting despite short-term fluctuations. OTU-level surveys over larger regions, enabled by high-throughput versions of this approach, could provide a clearer understanding of LSV dynamics in the managed honey bee population.

The paired thorax and abdomen samples were not uniform in terms of either detection pattern or metagenetic composition, which suggests these tissues may frequently differ in LSV abundance or strain composition. Technical variation in sequence amplification, particularly at lower viral abundance, could also contribute to these results, although a single high-diversity sample had low technical variation in our hands. Daughenbaugh et al. (2015) previously reported significantly different LSV2 abundances across body segments, but LSV strain diversity by tissue has not been investigated to our knowledge. Variation in viral abundance by tissue may be common among honey bee viruses (e.g., Fujiyuki et al., 2006) and can relate to disease presentation (Yue & Genersch, 2005), but there remain few data on differential tissue tropism of viral variants in honey bees. Differential tissue tropism is a potential outcome of quasispecies dynamics (Domingo, Sheldon & Perales, 2012) but might also exist among ecologically distinct viral lineages, such as proposed for the ‘Kakugo’ variant of the Deformed wing virus complex (Fujiyuki et al., 2006).

An interesting finding of this study was that the sweat bee *H. ligatus* had a similar per-individual detection rate as co-distributed *A. mellifera*, sampled as foragers on flowers in the vicinity of an urban apiary. Dolezal et al. (2016) encountered a comparable rate of positives in a sample of 32 *Halictus* (two of 32, 6.3%), and in 50 total Halictidae samples (7 of 50, 14%). Those authors also reported similar LSV abundance in infected individuals of these two bee families. Further metagenetic sequencing of co-distributed *A. mellifera* and *H. ligatus* could help assess the frequency of interspecies transfer of LSV as well as evaluate species-specific virulence and evolutionary responses to passaging experiments. As *H. ligatus* is primitively eusocial, comparisons of LSV dynamics within colonies of the two species might also be tractable. Interestingly, a novel viral group related to LSV was recently identified by metagenomic sequencing of a congener, *H. scabiosae* (Bigot et al., 2017), suggesting that Sinaivirus-like entities have diversified during bee evolution.

Assessing OTU diversity in samples is challenging, as it can be difficult to distinguish distinct viral strains from the spectrum of mutations arising post-infection, particularly in error-prone RNA viruses (Domingo, Sheldon, & Perales, 2012). Technical noise such as sequence error, chimeras, and coverage disparities also complicate diversity assessments (Hill et al., 2003). In this study, we were reluctant to employ common microbial diversity measures due to a lack of empirical justification for their use. We instead focused on identifying the proportion of OTUs within samples that were a given number of protein substitutions away from the most common OTU. We found that many samples contained a substantial proportion of OTUs that diverged by 15 amino-acid substitutions or more from the major haplotype. We view changes of this magnitude, at this proportion of the total LSV composition, as most likely co-infections by diverse LSV strains. For comparison, the LSV1 and LSV2 genome references of Runckel et al. (2011) differ by 23 residues within the 105-residue alignment (after primer trimming). That individuals and not merely colonies are co-infected is indicated by the UNL sample set: individual specimens collected as foragers harbored LSV compositions that were as or more diverse by this measure than the USGS and USDA pools (20 and 50 workers, respectively) collected from hives (Fig. 3). The reasons for this diversity are unclear and may only reflect the background distribution of LSV strains at the disjunct locations sampled. Infection dynamics within the hive could also contribute to compositional divergence between (older) foragers and (younger) nurse bees, but it would be necessary to confirm this with longitudinal studies of individual colonies. Regardless, the diversity of LSV strains observed in individual specimens appears to require frequent co-infection. Given that LSVs found in multiple other hymenoptera (Ravoet et al., 2015; Dolezal et al., 2016; Bigot et al., 2017; this study) are not obviously distinct from those found in *A. mellifera*, we speculate that LSVs retain a basal level of infectivity to a wide range of hymenopterans, perhaps achieving high abundance only in stressed hosts.

At present there is no evidence that LSV strains differ in their impact on managed bees or that LSV abundance is a concern for apiculture. Thus, a general assessment of LSV prevalence or relative abundance will likely be preferred for ongoing surveillance, i.e., samples pooled at the management unit (colony or apiary) as part of multi-pathogen surveys. Agricultural resource managers will likely continue to relate LSV loads to factors known to influence bee pathogens generally (e.g., season, transport and other stressors, co-distributed pests and pathogens, and host genetic background). The primers tested here should considerably simplify such surveys while providing potentially more consistent assessments of LSV abundance in the face of high genetic diversity.

## Conclusions

The genetic marker described here should simplify and strengthen future surveys of LSV prevalence, which is a common target in studies of honey bee health. Metagenetic sequencing of the amplicon should aid the discovery and experimental validation of genetic variation associated with host range, virulence, transmission, or other aspects of Sinaivirus ecology. While this metagenetic approach cannot uncover variation elsewhere in the viral genome, it can guide the selection of samples and methods for genome-level analysis, such as via shotgun, long-read, or sequence-capture methods. Moreover, the amplicon appears sufficiently diverse to “tag” all but the most closely related phylogenetic clusters, barring recombination.

##  Supplemental Information

10.7717/peerj.9424/supp-1 Figure S1 Visualization of the Lake Sinai Virus assay product by electrophoresis Amplicons were migrated in 1.2% agarose. Positive samples are individual bee specimens collected from Lincoln, Nebraska. Lane 1: 100 bp deoxyribonucleic acid ladder/marker, Lane 2–5: samples scored negative, Lane 6: tick markers, Lane 7–10: samples scored positive, Lane 11: Negative control.

10.7717/peerj.9424/supp-2 Figure S2 Pairwise correlations between technical replicates of sample UNL.VT.250 Scatterplot points represent the frequency of individual OTUs in each pair. Technical replicates are based on independent amplifications and library preparations from the same source sample.

10.7717/peerj.9424/supp-3 File S1 Lake Sinai Virus nucleotide alignment used for primer selection Bases are colorized to indicate overall conservation within the alignment. Location and orientation of each primer are indicated by arrows. Sequences were obtained from Cornman (2018).

10.7717/peerj.9424/supp-4 File S2 Script used to tabulate LSV diversity within samples The distribution of protein differences (unit increments) weighted by OTU frequency and relative to the most abundant OTU in each sample. The data files used as input are appended and commented out. The script is specific to the analysis described in the text and is not intended as a general tool.

10.7717/peerj.9424/supp-5 File S3 Example sequences obtained from positive LSV assay amplicons Sanger sequences from the USDA set. Maryland and California samples begin with MD and CA, respectively. F and R designate sequences obtained with forward and reverse primers, respectively. Sequence names indicate whether the sample was positive using LSV2-specific primers using the assay described in Traynor et al. (2016).

10.7717/peerj.9424/supp-6 File S4 Representative sequences of Lake Sinai Virus operational taxonomic units (OTUs) OTU clustering was performed at 98% sequence identity and denoised as described in the text. Sequence IDs are alphanumeric identifiers based on the underlying read from which the cluster representative derives.

10.7717/peerj.9424/supp-7 File S5 Raw occurrence counts of each Lake Sinai Virus OTU in each sample

## Additional Information and Declarations

### Field Study Permissions

The following information was supplied relating to field study approvals (i.e., approving body and any reference numbers): No new sampling occurred for this study. Sites used for sampling are listed in the cited previous work. These consisted of both our own institutional sites as well as private and public lands. Private property was accessed with the expressed verbal permission of landowners and beekeepers. As the location and health status of colonies is protected trade information, access is typically given on condition of anonymity and cannot be disclosed to other parties. However, we are authorized to state that access to apiaries on private lands in North Dakota were given by Zac Browning, Browning Honey Company. Access to apiaries on private lands in South Dakota were given by Brett Adee, Adee Honey Farms.

### Data Availability

The following information was supplied regarding data availability: OTU clusters and abundances, Sanger-sequenced amplicons, and code are available as Supplemental Files. Raw Illumina FASTQ reads and associated metadata are available at NCBI: PRJNA608977.
