# Supplementary figures and images for: An updated genetic marker for detection of Lake Sinai Virus and metagenetic applications
Source: PeerJ. 2020 Jul 17;8:e9424. doi: 10.7717/peerj.9424 (PMC7370930; doi:10.7717/peerj.9424)

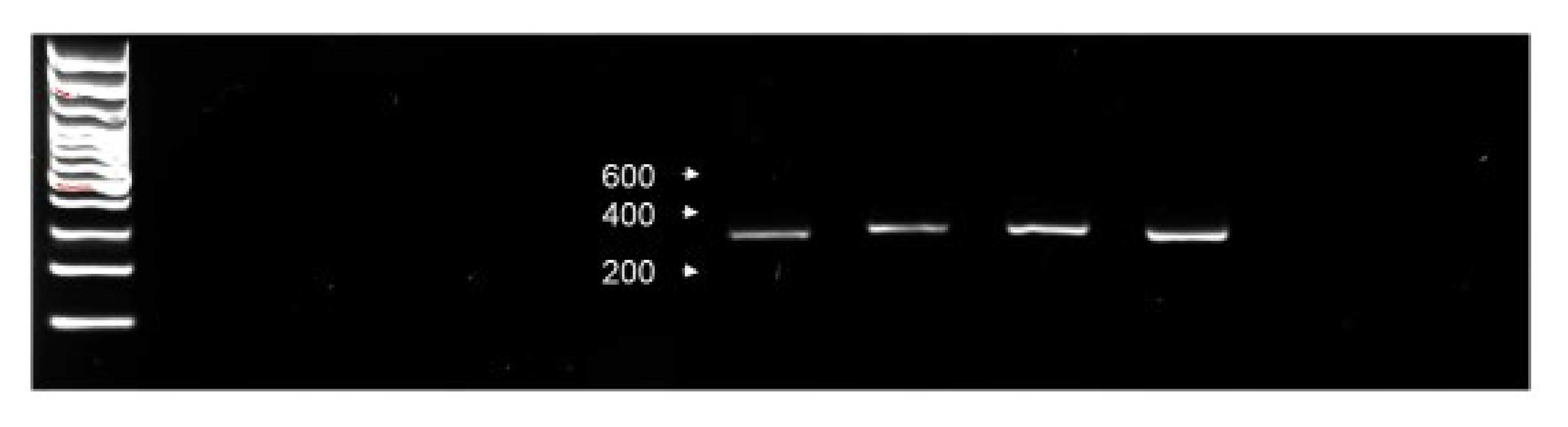

Supplement: Figure S1 — Amplicons were migrated in 1.2% agarose. Positive samples are individual bee specimens collected from Lincoln, Nebraska. Lane 1: 100 bp deoxyribonucleic acid ladder/marker, Lane 2–5: samples scored negative, Lane 6: tick markers, Lane 7–10: samples scored positive, Lane 11: Negative control. [file peerj-08-9424-s001.png]

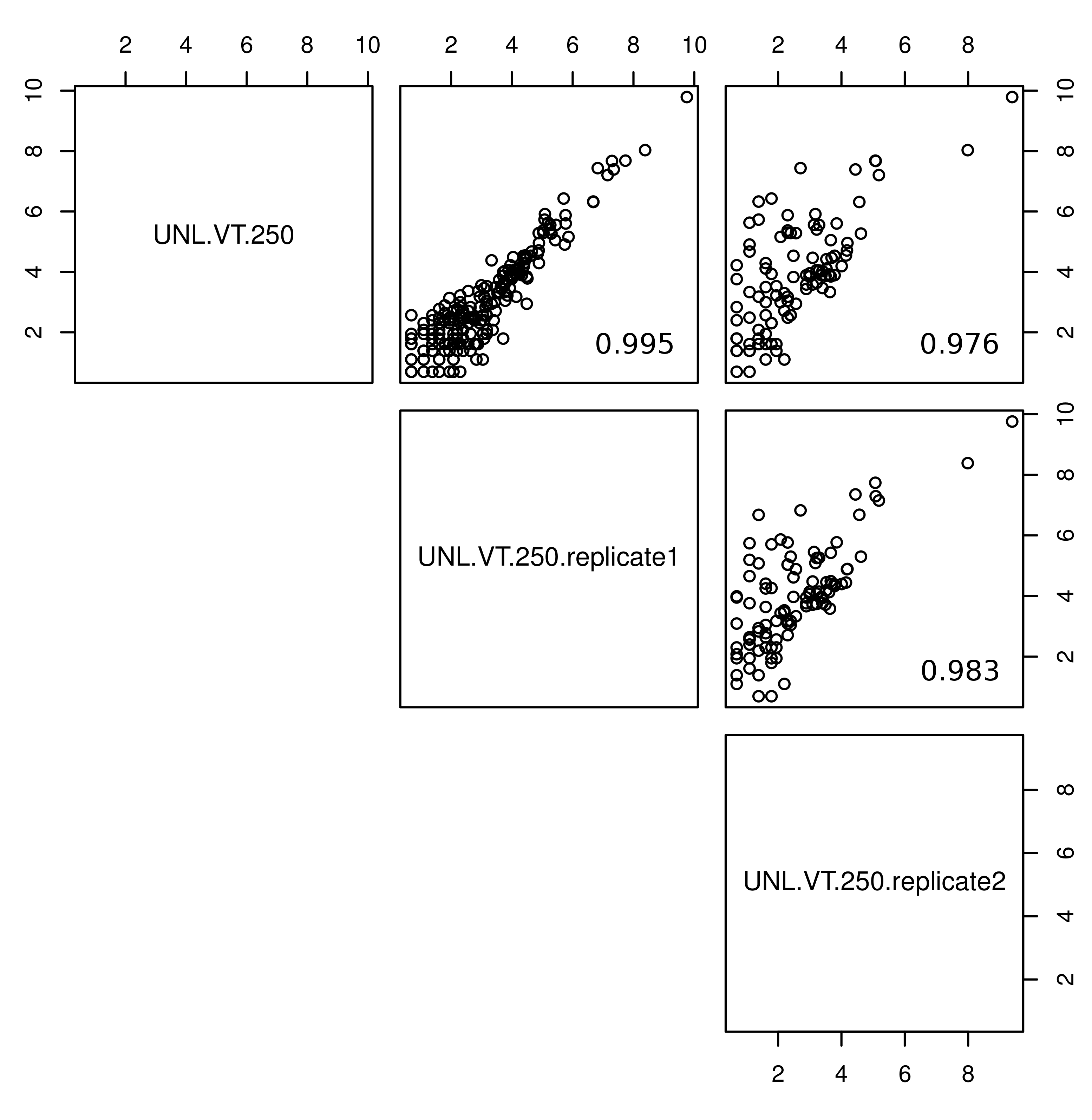

Supplement: Figure S2 — Scatterplot points represent the frequency of individual OTUs in each pair. Technical replicates are based on independent amplifications and library preparations from the same source sample. [file peerj-08-9424-s002.png]

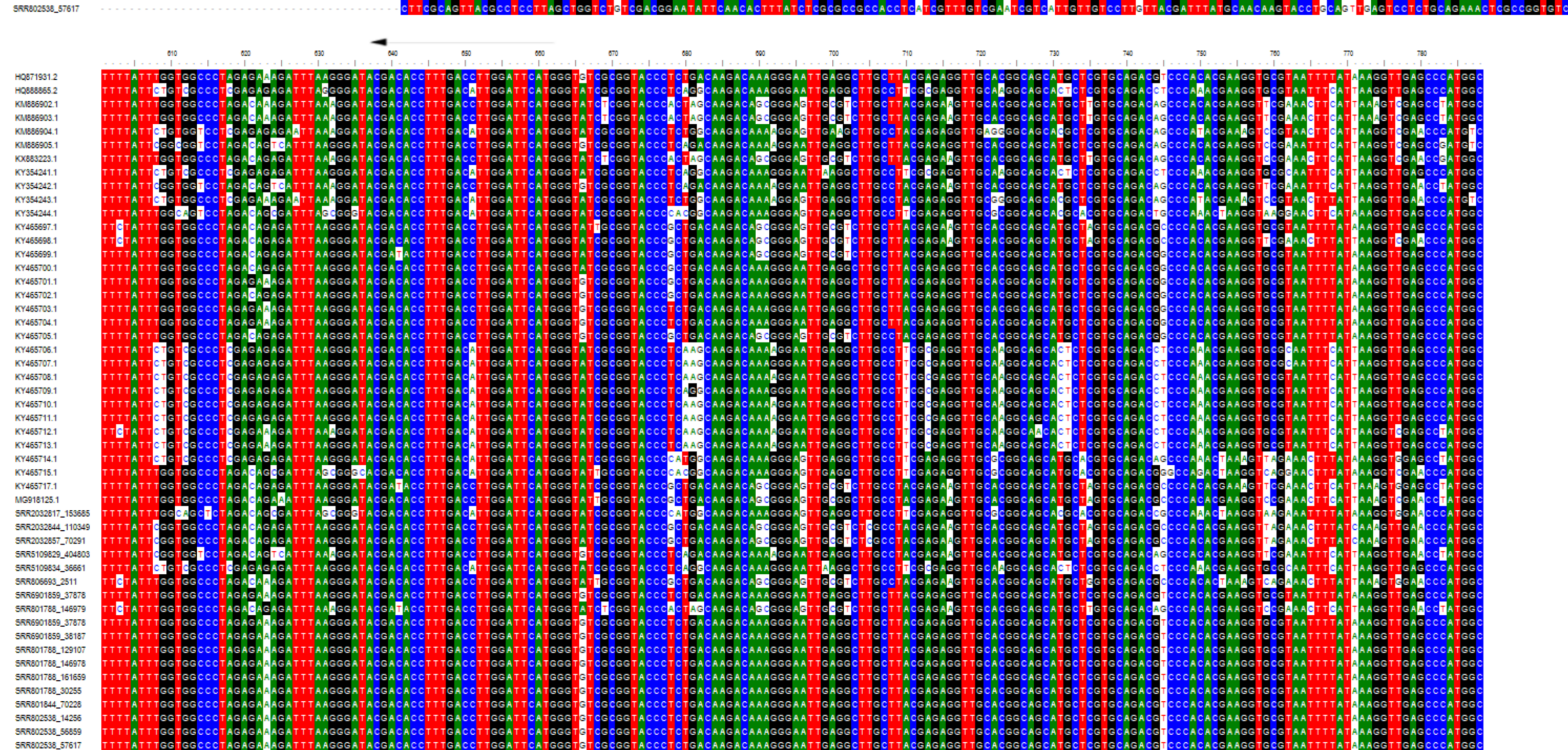

Supplement: File S1 — Bases are colorized to indicate overall conservation within the alignment. Location and orientation of each primer are indicated by arrows. Sequences were obtained from Cornman (2018). [file peerj-08-9424-s003.pdf]
